# Role of Galectin in Cardiovascular Conditions including Cirrhotic Cardiomyopathy

**DOI:** 10.3390/ph16070978

**Published:** 2023-07-07

**Authors:** Hongqun Liu, Sang-Youn Hwang, Samuel S. Lee

**Affiliations:** 1 Liver Unit, University of Calgary Cumming School of Medicine, Calgary, AB T2N 4N1, Canada; hliu@ucalgary.ca (H.L.); mongmani@daum.net (S.-Y.H.); 2 Department of Internal Medicine, Dongnam Institute of Radiological & Medical Sciences, Busan 46033, Republic of Korea

**Keywords:** galectin, lectin, pathophysiology, cardiac, cirrhotic cardiomyopathy, treatment

## Abstract

Abnormal cardiac function in the setting of cirrhosis and in the absence of a primary cardiac disease is known as cirrhotic cardiomyopathy. The pathogenesis of cirrhotic cardiomyopathy is multifactorial but broadly is comprised of two pathways. The first is due to cirrhosis and synthetic liver failure with abnormal structure and function of many substances, including proteins, lipids, hormones, and carbohydrates such as lectins. The second is due to portal hypertension which invariably accompanies cirrhosis. Portal hypertension leads to a leaky, congested gut with resultant endotoxemia and systemic inflammation. This inflammatory phenotype comprises oxidative stress, cellular apoptosis, and inflammatory cell infiltration. Galectins exert all these pro-inflammatory mechanisms across many different tissues and organs, including the heart. Effective therapies for improving cardiac function in patients with cirrhosis are not available. Conventional strategies for other noncirrhotic heart diseases, including vasodilators, are not feasible because of the significant baseline vasodilation in cirrhotic patients. Therefore, exploring new treatment modalities for cirrhotic cardiomyopathy is of great importance. Galectin-3 inhibitors such as modified citrus pectin, N-acetyllactosamine, TD139 and GB0139 exert anti-apoptotic, anti-oxidative and anti-inflammatory effects and thus have potential therapeutic interest. This review briefly summarizes the physiological and pathophysiological role of galectin and specifically examines its role in cardiac disease processes. We present a more detailed discussion of galectin in cardiovascular complications of cirrhosis, particularly cirrhotic cardiomyopathy. Finally, therapeutic studies of galectin-3 inhibitors in cirrhotic cardiomyopathy are reviewed.

## 1. Introduction

Cirrhosis is associated with a host of cardiovascular abnormalities. These include peripheral vasodilation with hyperdynamic circulation, portal hypertension, hepatopulmonary syndrome, and a condition called cirrhotic cardiomyopathy. This term was coined by Lee in 1989 [1] and has been the major focus of our translational research lab since then. It is defined as abnormal cardiac function in cirrhosis in the absence of any pre-existing primary heart disease [2]. Numerous factors associated with cirrhosis appear to play a pathogenic role, and this has been the topic of recent reviews [3,4,5,6,7]. Although several pathogenic mechanisms responsible for cirrhotic cardiomyopathy have been published by our lab and others, only recently have we clarified in a comprehensive review [4] the distinct dual pathways underlying these myriad mechanisms. Specifically, there are a group of pathogenic factors caused by liver failure with the inadequate or abnormal synthetic function of several substances, including proteins such as the cardiac contractile protein myosin heavy chain as well as lipids, lipoproteins, hormones, and carbohydrates such as lectins. The other pathway is related to portal hypertension with a congested gut and high mesenteric venous pressure. In this setting, bacterial translocation through the gut wall occurs, with endotoxemia and an inflammatory phenotype characterized by cytokine release, oxidative and nitrosidative stress, and inflammatory cell infiltration.

Galectins are members of the lectin family that derive their name from their ability to bind β-galactoside sugars. Galectin-3 is one of the 15 mammalian galectins identified to date [8]. It is a 30 kD protein encoded by the LGALS3 gene [9]. Galectin-3 is found in the nucleus and cytoplasm, at the cell surface, and is also secreted to the extracellular space allowing it to enter the blood circulation [10]. It has pleiotropic functions affecting cell adhesion processes [11], cell differentiation, fibrogenesis, inflammation [12], oxidative stress, and apoptosis [13]. It is well known that inflammation and fibrosis are pivotal factors and play pathophysiological roles in the development and progression of many kinds of heart diseases, including atrial fibrillation [14], heart failure [15], and cardiomyopathy [13,16].

The evidence that galectin-3 plays a role in the pathogenesis of cirrhotic cardiomyopathy is as follows: (1) galectin-3 levels are significantly increased in the circulation of patients with chronic heart failure, independent of etiology [17]. (2) Galectin is derived mainly from monocytes/macrophages, and our previous study showed that monocytes/macrophages are increased in cirrhotic hearts [18]. (3) Galectin-3 is a pro-inflammatory factor that stimulates TNFα production in a dose- and time-dependent manner [19]; we previously confirmed the pathogenic importance of TNFα in cirrhotic cardiomyopathy [20,21,22,23]. (4) Galectin-3 upregulates collagen I and increases the collagen I:III ratio [24], a phenomenon that has been demonstrated to occur in cirrhotic cardiomyopathy [20]. This plays an important role in the pathogenesis of diastolic dysfunction in the cirrhotic heart [25]. (5) Serum galectin-3 levels are associated with heart failure with a preserved ejection fraction (HFpEF) [26], and cirrhotic cardiomyopathy is an example of this type of heart failure [5].

The present review aims to summarize the role of galectin-3 in non-cirrhotic cardiac diseases and cirrhotic cardiomyopathy and to explore whether it may be a therapeutic target in cirrhotic cardiomyopathy.

## 2. Galectin in Various Cardiovascular Diseases

The question arises whether an increase in galectin-3 results from heart failure or results in heart failure. To address this question, Sharma et al. injected recombinant galectin-3 into the pericardial sac of healthy Sprague-Dawley rats and observed that it induced not only excess cardiac collagen deposition but also heart failure [24]. In the same study, using homozygous transgenic TGRmRen2-27 (Ren-2) rats, a heart failure-prone hypertensive model, these authors demonstrated that galectin-3 was upregulated in the heart following the development of heart failure. Moreover, galectin-3 is also significantly increased in hypertrophied hearts of patients with aortic stenosis [24]. Galectin-3 is also increased in drug-induced heart injuries, such as after administration of interferon γ [27], doxorubicin [13], streptozotocin [28] and angiotensin II [29]. All these studies indicate that galectin-3 plays a key pathogenic role in cardiovascular diseases.

Galectin-3 that insults the heart comes mainly from macrophages and is also derived from other cardiac cells, such as cardiomyocytes and fibroblasts in disease settings [30]. Galectin-3 is increased in different etiologies of heart disease. Nguyen et al. [31] measured the cardiac and plasma galectin-3 in several models of heart diseases in mice and demonstrated that both plasma and heart galectin-3 levels were increased in mice with fibrotic cardiomyopathy, ischemia-reperfusion-induced heart disease, and overactivation of β-adrenergic receptor-induced cardiomyopathy. Interestingly, the density of cardiac inflammatory cells was also significantly elevated in these animals. 

Another cardiac condition associated with increased plasma galectin-3 is acute myocardial infarction [14]. However, in a mouse model of dilated cardiomyopathy, the plasma galectin-3 levels were not increased, although the cardiac content of galectin-3 was increased 50-fold. This finding suggests that cardiac inflammation is a prerequisite for releasing galectin-3 into the circulation. Dilated cardiomyopathy lacks myocardial inflammatory infiltration, and therefore the plasma galectin-3 levels were not increased in patients with hypertrophic cardiomyopathy or dilated cardiomyopathy if there was no concomitant renal dysfunction.

In summary, most injuries to the heart trigger pro-inflammatory factors, including galectin-3, that produce further damage through an amplifying cascade. The increase in galectin-3 in the circulation is due to cardiac inflammation, which stimulates the leakage of galectin-3 from various cells into the circulation.

## 3. Galectin-3 and Cardiac Inflammation

As previously noted, galectin-3 is a pro-inflammatory factor [32]. Humphries et al. used lipopolysaccharide or bleomycin to induce lung inflammation and demonstrated that galectin-3 is increased in parallel along with the inflammation score and pro-inflammatory cytokines/chemokines such as IL-6, IL-8 and TNFα. GB0139, an inhibitor of galectin-3, significantly decreased inflammation severity, reduced the inflammation score, and levels of IL-6, IL-8, TNFα and macrophage inflammatory protein-1-alpha. This data implies that there is a causal correlation between galectin-3 and inflammation [32].

The possible mechanisms underlying galectin-induced inflammation include the following: (1) inflammatory cytokines: galectin-3 upregulates the expression of inflammatory cytokines and activates macrophages, promoting regional and systemic inflammation commonly seen in settings of heart disease [31]. (2) Toll-like receptor 4 (TLR4) is a transmembrane protein. Its activation further activates the NF-κB intracellular signaling pathway and inflammatory cytokines. Li and colleagues [33] showed that the blockade of galectin-3 significantly inhibits TLR4 expression in acute and chronic myocardial injury. (3) Oxidative stress and apoptosis: oxidative stress, apoptosis, and inflammation generally occur together in organs, including the heart [34]. Galectin-3 in the extracellular space plays a role in cell migration and cell-cell interactions and intracellularly regulates cell cycle and apoptosis [35]. 

Zong et al. [36] reported that lipopolysaccharide treatment increases galectin-3 expression in HMC3 cells (human microglial cell line), and oxidative stress is also increased. TD139, a specific galectin-3 inhibitor, significantly reduces galectin-3 expression and oxidative stress of HMC3 cells [34]. Furthermore, galectin-3 knockdown reduces oxidative stress and apoptosis in ARPE-19 cells (spontaneously arising retinal pigment epithelial cell line) [37]. It is known that oxidative stress and apoptosis are pathophysiological features of cardiac dysfunction [38]. Galectin-3 inhibition decreases apoptosis and oxidative stress in the heart and improves cardiac function [39]. 

## 4. Galectin-3 and Organ Remodeling/Fibrosis

Galectin-3 is involved in the fibrogenesis of many organs, such as the liver [40], kidney [41], and heart [42,43,44]. Henderson and colleagues [40] compared liver fibrogenesis in galectin-3^−/−^ and wild-type (WT) mice. They first demonstrated that galectin-3 expression is up-regulated in the fibrotic livers of human patients, then explored the mechanisms of galectin-3 on fibrogenesis. They documented that galectin-3 is co-localized with proliferating fibroblasts in carbon tetrachloride (CCl_4_)-treated mice. Eight weeks after CCL_4_ administration, the expression of α-SMA (smooth muscle actin), a marker of fibrosis, was significantly increased in wild-type compared with Galectin-3^−/−^ mice. Galectin-3^−/−^ mice had milder liver fibrosis after CCl_4_ administration compared with wild-type animals. Culture of hepatic satellite cells (HSCs) demonstrated that spontaneous HSC activation occurred in wild-type but not Galectin-3^−/−^ HSCs. After 7 days, the protein expression of α-SMA was significantly lower in Galectin-3^−/−^ HSCs compared with wild-type HSCs, and exogenous recombinant murine Galectin-3 significantly reversed the α-SMA expression in Galectin-3^−/−^ HSCs. All the data above demonstrated that Galectin-3 plays a central role in the activation of hepatic stellate cells and liver fibrosis. 

This phenomenon is also verified in the kidney [40,41]. Ou and colleagues demonstrated that plasma galectin-3 was negatively correlated with estimated glomerular filtration rate and positively correlated with renal fibrosis [41].

Galectin-3 plays a central role in cardiac remodeling [45]. Sharma et al. [24] showed that the increase of galectin-3 precedes the development of heart failure in an animal model of failure-prone hypertrophic cardiomyopathy. Recombinant galectin-3 has the capability to induce cardiac fibroblast proliferation and collagen synthesis. Galectin-3 directly increases the ratio of cardiac collagen I and collagen III [24]. Increased galectin-3 expression augments fibroblast activity, accumulation of extracellular matrix [24] and the synthesis of collagen production in the myocardium. Neutralization of Galectin-3 mitigates fibrosis, left ventricular dysfunction, and the development of heart failure [46]. Galectin-3 knockout mice are completely resilient to established pro-fibrotic perturbations, and cardiac fibrosis is almost completely absent in these mice [46].

## 5. Galectin Pathophysiology in Noncirrhotic Cardiovascular Conditions

Besides fibrogenesis, another effect of galectin-3 is the inhibition of cardiac function [47]. Van Kimmenade and colleagues [48] demonstrated a correlation between galectin-3 and heart failure. Wu et al. [47] evaluated the role of galectin-3 in the severity of diastolic cardiac dysfunction and found that the higher the plasma galectin-3 levels, the more severe the diastolic heart failure. Furthermore, plasma galectin-3 levels showed positive correlations with diastolic echocardiographic parameters (E/e’; r = 0.65; *p* < 0.001), extracellular volume fraction (r = 0.59; *p* = 0.002), and peak ejection rate (r = 0.59; *p* = 0.003) and a negative correlation with peak filling rate (r = −0.58; *p* = 0.003). 

Another study showed [49] that galectin-3 correlated significantly with both left ventricular end-systolic volume index and end-diastolic volume index. An effect of galectin-3 inhibitors on non-cirrhotic heart diseases is the attenuation of collagen deposition [50]. Sharma [24] et al. performed a microarray analysis of hearts from Ren-2 rats which are prone to develop hypertrophic heart failure. They showed that galectin-3 is the dominantly overexpressed gene in those animals that went on to develop overt heart failure. In addition, they infused galectin-3 directly into the pericardial cavity of healthy rats and found that it depressed left ventricular ejection fraction, fractional shortening, and the scale of the negative slope of dP/dt_max_. It also increased the levels of cardiac collagen I, the collagen I:III ratio, and the lung weight to body weight ratio. As collagen I is more rigid than collagen III, an increased I:III ratio leads to a stiffer, noncompliant ventricle and, thus, diastolic dysfunction. 

N-acetyl-seryl-aspartyl-lysyl-proline (Ac-SDKP) is a natural tetrapeptide and has anti-inflammatory and antifibrotic properties. Liu et al. [51] demonstrated that the co-infusion of galectin-3 and Ac-SDKP into the pericardial cavity inhibited fibrosis and inflammation and improved cardiac function. The inhibition of galectin-3 by N-Lac attenuates cardiac fibrosis and improves left ventricular function and subsequent heart failure [46]. 

## 6. Galectin in Cirrhotic Cardiomyopathy 

Galectin-3 levels are significantly increased in cirrhotic patients [52] and animal models of liver fibrosis [53]. Moreover, inflammation is part of the phenotype of cirrhotic cardiomyopathy: there are significant increases in pro-inflammatory cytokines such as TNFα [22], interleukin 1β [23], increased oxidative stress [21] and cardiomyocyte apoptosis [54]. 

We previously demonstrated an increase in the collagen I:III ratio in the cirrhotic murine heart, wherein the amount of the more rigid collagen I is significantly increased [20,25]. The long-term application of galectin-3 inhibitor decreases the synthesis of collagen I in the cirrhotic heart and improves cardiac contractility. Thus, these factors may play essential pathogenic roles in cardiac dysfunction in both non-cirrhotic and cirrhotic cardiomyopathy. 

Another consideration is the possible pathogenic role of galectin in atrial fibrillation. It is interesting that atrial fibrillation is the most common arrhythmia found in patients with cirrhosis [55,56,57]. The prevalence of atrial fibrillation in cirrhotic patients ranges between 6.6% and 14.2% [55]. The relationship between high serum galectin-3 and atrial fibrillation is still not entirely settled [14,58]. However, the majority of pertinent studies support the view that high serum galectin-3 is indeed associated with atrial fibrillation [14,55,57,59,60]. 

Atrial fibrillation often occurs in the setting of liver transplant surgery and is the most common arrhythmia in the perioperative period and the postoperative period after transplantation [61]. Indeed, atrial fibrillation is the dominant cardiovascular complication associated with liver transplantation and is a key feature of so-called ‘MACE’ (major adverse cardiovascular event) syndrome following transplant [61,62]. However, the underlying mechanisms remain unclear. The increased galectin in serum and heart may play a role in atrial fibrillation in cirrhotic patients. This arrhythmia has been associated with a variety of adverse outcomes [63], such as stroke, acute kidney injury and in-hospital mortality. Although the potential relationship between galectin and atrial fibrillation has not been studied to date in animal models or patients with cirrhosis, it is an intriguing potential cross-link that needs to be further explored.

**Figure 1:**
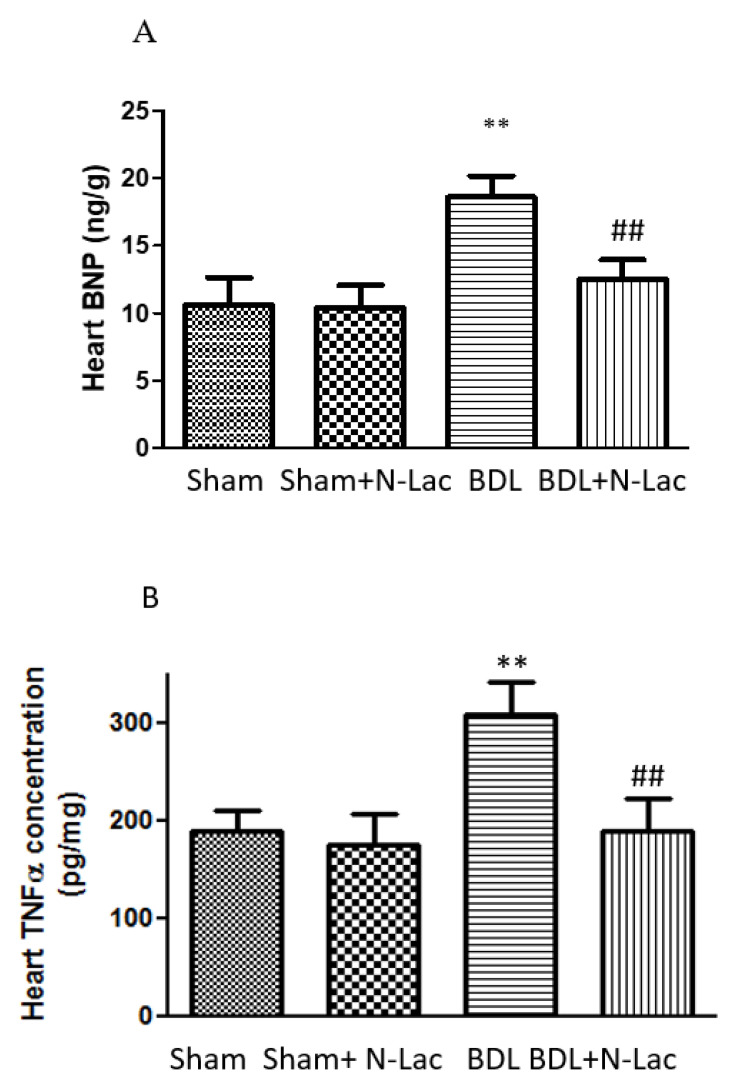
Heart BNP and TNFα levels. (**A**) BNP expression is significantly increased in BDL rats. N-Lac significantly decreased BNP concentration. (*n* = 6 in each group, ** *p* < 0.05 compared with sham; ## *p* < 0.05 compared with BDL). (**B**) TNFα expression is significantly increased in BDL rats; N-Lac significantly decreased TNFα concentration. (*n* = 6 in each group, ** *p* < 0.05 compared with sham; ## *p* < 0.05 compared with BDL). BNP: brain natriuretic peptide; TNFα: tumor necrosis factor-alpha; BDL: bile duct ligation. Adapted from reference [20]: Yoon KT et al., Clin Mol Hepatol 2022;28:232–241.

We recently examined a possible pathogenic role of galectin in cirrhotic cardiomyopathy [20] using a rat model of cirrhosis due to bile duct ligation (BDL). The rats were divided into 4 groups: sham-operated controls, sham + N-acetyllactosamine (N-Lac, an inhibitor of galectin-3), BDL and BDL + N-Lac. Cardiac galectin-3 was significantly increased in the BDL-cirrhotic rats. But cardiac galectin-3 content was significantly lower in the BDL + N-Lac group compared with that in the BDL group (Figure 1). N-Lac had no effect on galectin-3 content in sham-control hearts. Brain natriuretic peptide (BNP) and TNFα were significantly increased in BDL-cirrhotic hearts compared to sham controls, and N-Lac significantly decreased galectin-3, TNFα, and BNP in cirrhotic hearts but not in sham-operated controls. In parallel with the decrease in TNFα and BNP, N-Lac significantly improved the systolic and diastolic contractility of cardiomyocytes from cirrhotic rats but had no effect in sham controls. 

**Figure 2:**
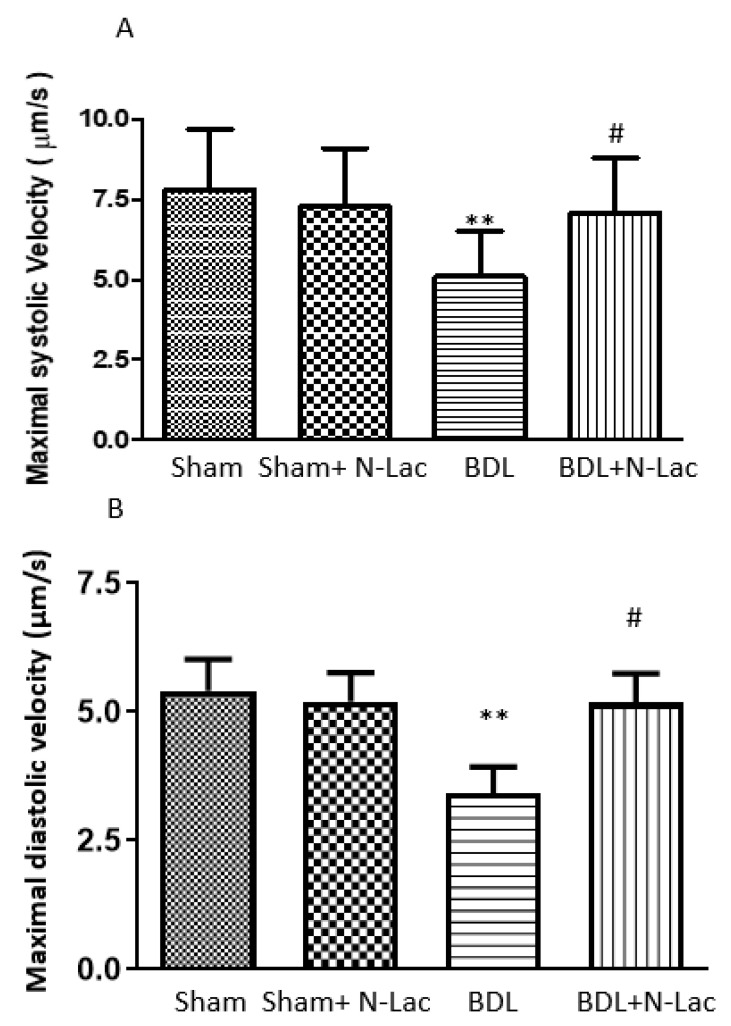
Isolated cardiomyocyte contractility and relaxation. (**A**) Isoproterenol-stimulated maximal systolic velocity. Systolic velocity was significantly decreased in BDL cirrhotic rats compared to the sham control group (** *p* < 0.05). Treatment of the BDL cirrhotic cells with N-Lac significantly reversed the reduced systolic contractility in this group (# *p* < 0.05 compared to the BDL group; *n* = 6 in each group). (**B**) Diastolic relaxation velocity under isoproterenol stimulation. Diastolic relaxation velocity was significantly decreased in BDL cirrhotic rat cells compared to the sham-control cells (** *p* < 0.01). Treatment of the BDL cardiomyocytes with N-Lac significantly reversed the reduced diastolic relaxation velocity in BDL rats (# *p* < 0.05 compared to the BDL group; *n* = 6 in each group). Sham: sham-operated; BDL: bile duct ligation; N-Lac: *N*-acetyllactosamine. Adapted from reference [20]: Yoon KT et al., Clin Mol Hepatol 2022;28:232–241.

BNP is a serum marker of heart failure or cardiac distress. The increase of BNP in the cirrhotic heart implies cardiac dysfunction in BDL rats. The decrease of cardiac BNP in the BDL + N-Lac group suggests that N-Lac improves cardiac function. Moreover, cardiac systolic and diastolic functions were significantly improved in the BDL + N-Lac group compared with that in BDL rats. Figure 2 shows β-adrenergic receptor agonist isoproterenol-stimulated maximal contractile and relaxation velocities in isolated perfused cardiomyocytes. We measured maximum sarcomere length change (dL) at certain time points (dt) to calculate the maximal contractile and relaxation velocities (dL/dt). Systolic and diastolic velocities were significantly decreased in cirrhotic rats compared to the sham control group. Treatment of the cirrhotic group with the galectin-3 inhibitor N-Lac significantly reversed the reduced systolic and diastolic velocities in cardiomyocytes from BDL cirrhotic rats (Figure 2). 

BNP and galectin-3 are associated with the severity of portal hypertension in cirrhotic cardiomyopathy. Abbas et al. [64] performed echocardiography and laboratory investigations, including BNP and galectin-3, in 71 patients (26 cirrhotic patients without ascites, 25 with ascites, and 20 healthy controls). They showed that BNP, galectin-3, and parameters of diastolic dysfunction were significantly increased according to the severity of portal hypertension and cirrhosis, with significant differences between the three groups. Moreover, they reported that both BNP and galactin-3 concentrations showed good sensitivity and specificity for the detection of diastolic dysfunction in cirrhotic patients (sensitivity: 77.6% and 95.5%, and specificity: 89.9% and 86.4%, respectively).

Another inflammatory cytokine that is involved in cardiac dysfunction in cirrhotic cardiomyopathy is TNFα. We previously demonstrated [22] that plasma TNFα is increased in BDL mice. Moreover, cardiac mRNA and protein expression of NFκB p65, p38 MAPK, iNOS, nitric oxide, and anandamide are increased in parallel with TNFα, and isoproterenol-stimulated cardiac contractility is blunted in isolated cardiomyocytes from BDL mice. Anti-TNFα antibody significantly decreases cardiac anandamide and nitric oxide, reduces the expression of NFκB p65, p38 MAPK, and iNOS, and improves systolic and diastolic velocities of cardiomyocytes from BDL mice compared with BDL controls. Accordingly, our previous study [20] demonstrated that TNFα plays an important role in the pathogenesis of cardiac dysfunction in BDL mice.

## 7. Galectin-3 Inhibitor to Treat Cirrhotic Cardiomyopathy

There is currently no well-established management strategy for cirrhotic cardiomyopathy. Theoretically, the therapeutic strategies for cardiac dysfunction in cirrhotic patients should be the same as those for non-cirrhotic heart failure, which include reducing cardiac preload and afterload, i.e., diuretic treatment and vasodilatation. Since cirrhotic cardiomyopathy is a long-term condition and long-term use of diuretics can be associated with renal injury, electrolyte disturbances and hepatorenal syndrome [65], diuretics are not the appropriate treatment for cirrhotic cardiomyopathy. Vasodilators used in non-cirrhotic cardiac dysfunction, such as angiotensin-converting enzyme (ACE) inhibitors or calcium channel blockers, may be hazardous to use in cirrhotic cardiomyopathy because these patients are already vasodilated and have low blood pressures [66]. Direct inotropic drugs such as cardiac glycosides are not effective in improving any contractile parameters [67]. Other agents that do not markedly affect peripheral circulation may therefore be useful in the management of cirrhotic cardiomyopathy.

**Figure 3:**
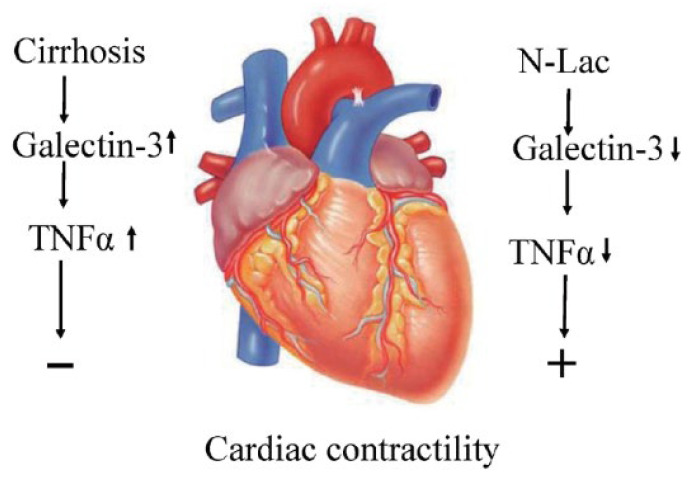
Schematic diagram: The elevated galectin-3 in a cirrhotic heart decreases cardiac contractility via the increase of TNFα; the galectin-3 inhibitor, N-Lac, reverses the inhibited cardiac contractility in a cirrhotic heart. TNFα: tumor necrosis factor-alpha; N-Lac: N-acetyllactosamine. (Reproduced from reference [20]: Yoon KT et al., Clin Mol Hepatol 2022;28:232–241).

Galectin-3 inhibitors seem to meet this condition. Galectin-3 is a modifiable factor in heart failure [45]. As aforementioned, galectin-3 has a stimulatory effect on inflammation, apoptosis, oxidative stress and fibrogenesis. Interestingly, all these factors co-exist in cirrhotic cardiomyopathy and therefore, galectin-3 inhibitors theoretically are attractive therapeutic options to explore in cirrhotic cardiomyopathy (Figure 3).

Yu et al. [46] showed that in wild-type mice, angiotensin II and transverse aortic constriction causes left ventricular hypertrophy, fibrosis, increased left ventricular end-diastolic pressure and decreased fractional shortening. In galectin-3 knock-out mice, although angiotensin II and transverse aortic constriction causes left ventricular hypertrophy, there were no left ventricular dysfunction and fibrosis. 

**Figure 4:**
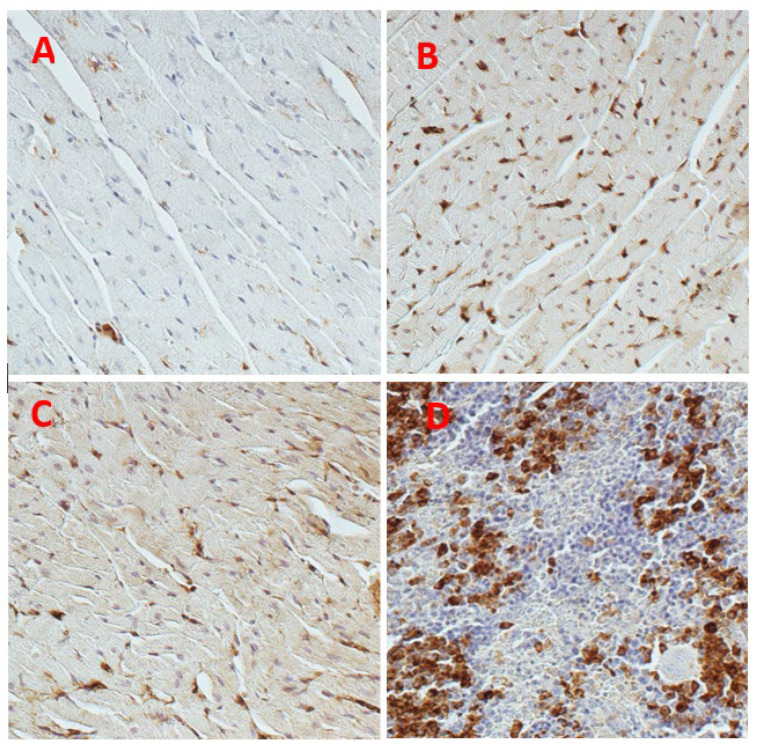
Representative immunohistochemistry analysis of galectin-3 protein expression in hearts (×200). (**A**) Sham. (**B**) BDL. (**C**) BDL + N-Lac. (**D**) Spleen as positive control. Computerized optical densitometry showed that galectin-3 expression was significantly increased in BDL; N-Lac significantly decreased galectin-3 expression in BDL. BDL: bile duct ligation; N-Lac, N-acetyllactosamine. (Reproduced from reference [20]: Yoon KT et al., Clin Mol Hepatol 2022;28:232–241).

Using isoproterenol (ISO)-induced cardiac hypertrophy in rats, Li et al. [33] demonstrated that hypertrophy-related genes, including atrial natriuretic peptide (ANP), brain natriuretic peptide (BNP), and β-myosin heavy chain (β-MHC), and the heart weight/body weight (HT/BT) ratio were increased, which was associated with significantly reduced cardiac function. Modified citrus pectin, a specific inhibitor of galectin-3, decreased the levels of ANP, BNP, and β-MHC, prevented cardiac hypertrophy, and improved cardiac function. These data suggest that modified citrus pectin may be a useful treatment for pathological cardiac hypertrophy. Another inhibitor of galectin-3 is N-Acetyl-D-lactosamine (N-Lac). Using a hypoxia-induced pulmonary arterial hypertension model in rats, Luo et al. [68] found that galectin-3 was significantly upregulated in pulmonary artery adventitia. Galectin-3 stimulated the excessive proliferation and differentiation of pulmonary adventitial fibroblasts and collagen synthesis. These effects of galectin-3 were reversed by N-Lac. As detailed in the previous section, N-Lac significantly improved the systolic and diastolic contractility in cardiomyocytes from cirrhotic rat hearts (Figure 4). 

There is also great interest in galectin inhibitors to treat nonalcoholic fatty liver disease (NAFLD). Belapectin (galactoarabino-rhamnogalacturonate [GRMD-02]) is also an inhibitor of galectin-3. Traber et al. reported that belapectin markedly reduced fibrosis, portal pressure, and septal galectin-3 positive macrophages in rats with cirrhosis [69]. Harrison et al. [70] performed a randomized, double-blind clinical study using belapectin in patients with biopsy-proven non-alcoholic steatohepatitis with advanced fibrosis. They showed that belapectin was safe and well tolerated, with evidence of a pharmacodynamic effect. They also reported that three of the five subjects treated with belapectin had a 20% reduction in liver stiffness measured by transient elastography (Fibroscan) compared to the baseline value, with two subjects having a reduction of ~50% from baseline. 

Based on these results, Chalasani et al. [71] performed a phase 2b double-blind, multicenter randomized clinical trial in 162 patients with biopsy-proven non-alcoholic steatohepatitis with cirrhosis and portal hypertension (hepatic venous pressure gradient ≥6 mmHg) to demonstrate safety and efficacy of belapectin. They assigned 162 patients in 36 medical centers evenly to three groups (placebo and biweekly infusions of belapectin at 2 mg/kg and 8 mg/kg) and treated patients for 52 weeks while monitoring changes in the hepatic venous pressure gradient and liver histology. They reported that the mean change of hepatic venous pressure gradient between the belapectin-treated groups and placebo group was not significantly different, although there was a tendency towards a slight reduction in the hepatic venous pressure gradient (−0.28 mgHg vs. 0.10 mmHg in 2 mg/kg belapetcin treated group, −0.25 mg vs. 0.10 mmHg in 8 mg/kg belapectin treated group). They also found that there was no significant difference in fibrosis or liver-related outcomes among the three groups. However, they showed that 2 mg/kg belapectin significantly reduced the mean hepatic venous pressure gradient (−1.16 mmHg vs. 0.4 mmHg, *p* = 0.02) and new development of esophageal varices (0% vs. 18%, *p*=.032) compared to placebo in the subgroup analysis of patients without oesophageal varices at baseline. Therefore, belapectin can be effective in improving portal pressure in patients with mild portal hypertension without esophageal varices. This implies that galectin-3 inhibitors may exert a beneficial therapeutic effect on cirrhotic cardiomyopathy because portal hypertension is an important mechanism of cirrhotic cardiomyopathy. Further randomized controlled studies using several inhibitors of galectin-3, including modified citrus pectin, N-Lac, and belapectin, should be considered in patients with cirrhotic cardiomyopathy.

## 8. Conclusions

Galectin-3 plays a central role in different types of cardiovascular diseases. The mechanisms of the therapeutic effect of galectin-3 inhibitors are via the inhibition of inflammation, oxidative stress, apoptosis, and fibrosis. There is no documented evidence that galectin-3 has an effect on hyperdynamic circulation in cirrhosis; therefore, galectin-3 inhibition mainly mediates its effects by reducing inflammation, oxidative stress, apoptosis and fibrosis in the cardiovascular system of cirrhosis. Accordingly, galectin-3 may be a novel potential target for the treatment of cirrhotic cardiomyopathy.

## Data Availability

Not applicable.
